# Biomarkers Predictive for In-Hospital Mortality in Patients with Diabetes Mellitus and Prediabetes Hospitalized for COVID-19 in Austria: An Analysis of COVID-19 in Diabetes Registry

**DOI:** 10.3390/v14061285

**Published:** 2022-06-13

**Authors:** Faisal Aziz, Hannah Stöcher, Alexander Bräuer, Christian Ciardi, Martin Clodi, Peter Fasching, Mario Karolyi, Alexandra Kautzky-Willer, Carmen Klammer, Oliver Malle, Felix Aberer, Erich Pawelka, Slobodan Peric, Claudia Ress, Caren Sourij, Lars Stechemesser, Harald Stingl, Thomas Stulnig, Norbert Tripolt, Michael Wagner, Peter Wolf, Andreas Zitterl, Othmar Moser, Christian Schelkshorn, Susanne Kaser, Harald Sourij, for the COVID-19 in Diabetes in Austria

**Affiliations:** 1 Interdisciplinary Metabolic Medicine Trials Unit, Department of Endocrinology and Diabetology, Medical University of Graz, 8036 Graz, Austria; faisal.aziz@medunigraz.at (F.A.); hannah.stoecher@stud.medunigraz.at (H.S.); oliver.malle@medunigraz.at (O.M.); felix.aberer@medunigraz.at (F.A.); norbert.tripolt@medunigraz.at (N.T.); 2 Medical Division for Endocrinology, Rheumatology and Acute Geriatrics, Wilhelminen Hospital Vienna, 1160 Vienna, Austria; alexander.braeuer@wienkav.at (A.B.); peter.fasching@gesundheitsverbund.at (P.F.); 3 Clinical Division for Internal Medicine, Endocrinology, Diabetology and Metabolic Diseases, St. Vinzenz Hospital Zams, 6511 Zams, Austria; christian.ciardi@krankenhaus-zams.at; 4 Clinical Division for Internal Medicine, Konventhospital Barmherzige Brüder Linz, 4020 Linz, Austria; martin.clodi@bblinz.at (M.C.); carmen.klammer@bblinz.at (C.K.); 5 4th Medical Division with Infectiology, SMZ Süd–KFJ-Hospital Vienna, 1100 Vienna, Austria; mario.karolyi@wienkav.at (M.K.); erich.pawelka@wienkav.at (E.P.); 6 Clinical Division for Endocrinology and Diabetology and Metabolic Diseases, AKH Vienna, 1090 Vienna, Austria; alexandra.kautzky-willer@meduniwien.ac.at (A.K.-W.); peter.wolf@meduniwien.ac.at (P.W.); 7 3rd Department and Karl Landsteiner, Institute for Metabolic Diseases and Nephrology, Clinic Hietzing, Vienna Health Care Group, 1130 Vienna, Austria; slobodan.peric@gesundheitsverbund.at (S.P.); thomas.stulnig@gesundheitsverbund.at (T.S.); andreas.zitterl@wienkav.at (A.Z.); 8 Department for Internal Medicine I, Medical University Innsbruck, 6020 Innsbruck, Austria; claudia.ress@i-med.ac.at (C.R.); susanne.kaser@i-med.ac.at (S.K.); 9 Clinical Division for Cardiology, Medical University Graz, 8036 Graz, Austria; caren.sourij@medunigraz.at; 10 Department for Internal Medicine I, Paracelsus Medical University, 5020 Salzburg, Austria; l.stechemesser@salk.at; 11 Division for Internal Medicine, Hospital Melk, 3390 Melk, Austria; harald@stingl.info (H.S.); michael.c.wagner@gmx.at (M.W.); 12 Department for Exercise Physiology & Metabolism, University of Bayreuth, 95445 Bayreuth, Germany; othmar.moser@uni-bayreuth.de; 13 Division for Internal Medicine, Hospital Stockerau, 2000 Stockerau, Austria; christian.schelkshorn@stockerau.lknoe.at

**Keywords:** COVID-19, SARS-CoV-2, diabetes mellitus, biomarker

## Abstract

Background: This study assessed the predictive performance of inflammatory, hepatic, coagulation, and cardiac biomarkers in patients with prediabetes and diabetes mellitus hospitalized for COVID-19 in Austria. Methods: This was an analysis of a multicenter cohort study of 747 patients with diabetes mellitus or prediabetes hospitalized for COVID-19 in 11 hospitals in Austria. The primary outcome of this study was in-hospital mortality. The predictor variables included demographic characteristics, clinical parameters, comorbidities, use of medication, disease severity, and laboratory measurements of biomarkers. The association between biomarkers and in-hospital mortality was assessed using simple and multiple logistic regression analyses. The predictive performance of biomarkers was assessed using discrimination and calibration. Results: In our analysis, 70.8% had type 2 diabetes mellitus, 5.8% had type 1 diabetes mellitus, 14.9% had prediabetes, and 8.6% had other types of diabetes mellitus. The mean age was 70.3 ± 13.3 years, and 69.3% of patients were men. A total of 19.0% of patients died in the hospital. In multiple logistic regression analysis, LDH, CRP, IL-6, PCT, AST-ALT ratio, NT-proBNP, and Troponin T were significantly associated with in-hospital mortality. The discrimination of NT-proBNP was 74%, and that of Troponin T was 81%. The calibration of NT-proBNP was adequate (*p* = 0.302), while it was inadequate for Troponin T (*p* = 0.010). Conclusion: Troponin T showed excellent predictive performance, while NT-proBNP showed good predictive performance for assessing in-hospital mortality in patients with diabetes mellitus hospitalized with COVID-19. Therefore, these cardiac biomarkers may be used for prognostication of COVID-19 patients.

## 1. Introduction

In late 2019, a novel coronavirus was discovered in Wuhan, China, which was later named COVID-19. COVID-19 spread rapidly around the globe and was declared a pandemic on 11 March 2020 [1]. As of 8 April 2022, there have been 494,587,638 confirmed cases of COVID-19 and 6,170,283 deaths worldwide [1]. At this time in Austria, there have been 3,998,130 cases of COVID-19 and 15,982 deaths [1].

Studies from China, Austria, and other European countries investigating the association between diabetes mellitus and COVID-19 have found that diabetes mellitus adversely affects the prognosis of COVID-19 via various mechanisms. Hyperglycemia increases inflammation by producing oxidative stress and consequently increases the risk of cytokine storm [2]. Hyperglycemia also supports viral proliferation via increased viral replication in monocytes [2]. The antiviral immune and inflammatory responses in infected patients with diabetes mellitus can also change insulin sensitivity by impairing glucose metabolism [2]. It has been reported that patients with diabetes mellitus do not contract COVID-19 infection more frequently than people without diabetes; however, a higher prevalence, a severe and prolonged course of the disease, and increased mortality have been shown in people with diabetes [3]. In this regard, studies have shown that diabetes is present in 17–33% of hospitalized COVID-19 patients [4,5,6]. Other studies have reported that patients with diabetes are 15% more likely to be admitted to critical care units [7], 2.6 times more likely to develop severe infection, and 2 times more likely to die compared with those without diabetes [8]. The above-described mechanisms of oxidative stress and pathophysiological cytokine production may also induce endothelial damage, which results in thromboembolic events and organ damage and therefore leads to higher complication rates [2].

As COVID-19 is considered a multisystem disease, studies have reported that various inflammatory, hepatic, coagulation, and cardiac biomarkers are correlated with COVID-19 disease severity and mortality. Specifically, elevated levels of CRP, procalcitonin, and interleukin-6 have been associated with increased mortality. Moreover, cardiac injury has been reported in 20% of patients with COVID-19; therefore, cardiac markers such as NT-proBNP and Troponin have also been found significantly elevated in those who died or had severe disease [9,10,11]. Furthermore, in patients with diabetes mellitus, D-dimer has been reported as a significant predictor of COVID-19 mortality [12], while CRP has been associated with a severe course of COVID-19 in patients [13]. However, evidence regarding the role of biomarkers in COVID-19 patients with diabetes mellitus is still scarce. Therefore, we assessed the predictive performance of various inflammatory, hepatic, coagulation, and cardiac biomarkers in patients with prediabetes and diabetes mellitus hospitalized for COVID-19.

## 2. Materials and Methods

### 2.1. Study Design

We performed a retrospective analysis of COVID-19 patients who are enrolled in the COVID-19 diabetes registry. The COVID-19 diabetes registry is an ongoing multicenter cohort study of diabetes mellitus patients who are hospitalized with confirmed SARS-CoV-2 infection in 11 participating hospitals in Austria. This study was initiated on 15 April 2020 and is sponsored by the Austrian Diabetes Association. For this analysis, data collected until 30 April 2021 were considered. The methodological details of this study are described in the study published by Sourij et al. [14].

### 2.2. Study Population and Inclusion Criteria

Both men and women aged ≥18 years with a positive throat swab for SARS-CoV-2 and a confirmed diagnosis of diabetes mellitus or prediabetes were enrolled in this analysis. Types of diabetes mellitus include type 1 diabetes mellitus, type 2 diabetes mellitus, and other types of diabetes mellitus. Diabetes mellitus was diagnosed according to the Austrian Diabetes Association criteria and prediabetes was defined as glycated haemoglobin (HbA1c) of 5.7–6.4% (39–46 mmol/mol), which was measured if glucose levels were elevated in patients without a known diagnosis of diabetes mellitus [15]. In the current data analysis, only patients hospitalized for COVID-19 were included.

### 2.3. Data Collection

Designated study coordinators and clinicians at each participating hospital collected pseudonymized data from eligible patients using an electronic case report form (CRF). Clinical variables were collected from the medical files of patients and the values of biomarkers and other laboratory measures were collected from the local clinical laboratory of each participating hospital.

### 2.4. Study Variables

#### 2.4.1. Outcome

The primary outcome of this study is in-hospital mortality, which is defined as death from the date of admission for COVID-19 to the date of discharge from the hospital.

#### 2.4.2. Predictors

The predictor variables were collected at the time of admission and comprised data related to demographic characteristics, clinical parameters, comorbidities, use of medication, course of the disease, and laboratory measurements.

Demographic characteristics included age and gender. Clinical data included weight, height, oxygen saturation, systolic and diastolic blood pressure, pulse, smoking status, and classification and duration of diabetes mellitus. Data related to the course of the disease included length of stay, ICU requirement, assisted ventilation, and specific COVID-19 therapy.

Comorbidities included heart failure, coronary heart disease (CHD), hypertension, central arterial occlusion disease, peripheral arterial occlusion disease, stroke, myocardial infarction, chronic kidney disease (CKD), autoimmune diseases, tumor diseases, chronic obstructive pulmonary disease (COPD), bronchial asthma, interstitial lung diseases, transplantations, and various liver diseases, such as non-alcoholic fatty liver disease (NAFLD) (biopsy-confirmed and patient-reported), congenital diseases (e.g., Mb. Wilson), viral hepatitis, cancer, and alcohol abuse.

The following oral antidiabetic drugs were charted: metformin, sulfonylureas, dipeptidyl-peptidase-4 inhibitors, sodium-dependent glucose co-transporter-2 inhibitors, and glucagon-like peptide receptor agonists. Insulin therapy was distinguished as premixed insulin, basal insulin, bolus insulin, and/or insulin pump therapy. Concomitant medications included blood-pressure-regulating drugs (angiotensin-converting enzyme inhibitors, angiotensin II receptor blockers, beta-blockers, calcium antagonists, central antihypertensives, thiazides, loop diuretics, mineral corticoid receptor blockers, and sacubitril), immune-regulating drugs (glucocorticoids and other immunosuppressants), anticoagulants, and pain medications (ibuprofen). Specific COVID-19 therapy was recorded as the use of antiviral, antibiotic, and antifungal drugs, corticosteroids, and anticoagulants.

Laboratory measurements included random glucose, HbA1c, plasma lipoproteins (total cholesterol, high-density lipoprotein cholesterol (HDL-cholesterol), low-density lipoprotein cholesterol (LDL-cholesterol), and triglycerides), hematological markers (leukocytes, hemoglobin, platelets), renal markers (estimated glomerular filtration rate (eGFR)), hemolysis parameter (lactate dehydrogenase (LDH)), liver enzymes (glutamate oxaloacetate transaminase/aspartate aminotransferase (GOT/AST), glutamate pyruvate transaminase/alanine aminotransferase (GPT/ALT), and gamma-glutamyl transferase (GGT)), inflammation markers (C-reactive protein (CRP), ferritin, procalcitonin (PCT), and interleukin-6 (IL-6)), coagulation markers (fibrinogen and D-dimer), and cardiac markers (N-terminal pro-B-type natriuretic peptide (NT-proBNP), and Troponin T).

### 2.5. Ethical Considerations

The study was approved on 15 April 2020 by the Ethics Committee of the Medical University of Graz, Graz, Austria (EK 32-355 ex 19/20) with subsequent amendments for countrywide data collection. Written informed consent was obtained from living patients to participate in the study, if possible. Patients who were unable to provide informed consent before their hospital discharge were contacted later to agree on the use of their clinical data. For patients who died before they could provide consent or where it was not possible to obtain consent, the Ethics Committee waived the need for informed consent. All data were anonymized prior to the analysis.

### 2.6. Statistical Analysis

For this study, data were extracted in Microsoft Excel format and analyzed in Stata version 17.0 and R studio version 2022.02.0. Categorical variables are described as frequencies with corresponding percentages (%). Quantitative variables are described as mean ± standard deviation (SD) or median with interquartile range [IQR] if the normal distribution was violated. Normality of quantitative variables was assessed using the Shapiro–Wilk test. Categorical variables were compared with in-hospital mortality using the Chi-square test. Quantitative variables were compared with in-hospital mortality using the unpaired t-test or their non-parametric equivalent Wilcoxon rank-sum test.

As all biomarkers were positively skewed, they were therefore log-transformed before further analysis. The correlation between each log-transformed biomarker was assessed using the Pearson correlation method and displayed as a scatterplot with the corresponding correlation coefficient and the *p*-value. The association between transformed biomarkers and in-hospital mortality was then assessed using simple and multiple logistic regression analyses. In simple logistic regression, the association of each biomarker was assessed individually with in-hospital mortality. In multiple logistic regression, the association of each biomarker with in-hospital mortality was adjusted for age, sex, and type of diabetes mellitus. The predictive performance of biomarkers was assessed using discrimination and calibration. Discrimination was measured in terms of concordance statistics (C-statistic) and displayed as an area under the receiver operator characteristic curve (AUC) plot. Calibration was measured by the Hosmer–Lemeshow goodness-of-fit test.

## 3. Results

### 3.1. Characteristics of Study Participants, Overall and by In-Hospital Mortality

The distribution of characteristics, comorbidities, and biomarkers of patients with diabetes mellitus hospitalized for COVID-19 are shown in Table 1. In total, 747 people were included in the analysis. The mean age was 70.3 ± 13.3 years. There were 518 (69.3%) males and 229 (30.7%) females enrolled in the analysis. The mean body mass index (BMI) was 29.0 ± 5.9 kg/m^2^. Most patients had type 2 diabetes mellitus (70.8%), followed by prediabetes (14.9%), other types of diabetes mellitus (8.6%), and type 1 diabetes mellitus (5.8%). Common comorbidities were hypertension (68.0%), coronary heart disease (26.5%), chronic kidney disease (21.4%), respiratory disease (19.7%), and peripheral artery disease (13.9%).

A total of 142 (19.0%) patients died in the hospital during the study period. Patients who died (78.6 ± 10.0 years) were significantly (*p* < 0.001) older compared to the survivors (68.3 ± 13.2 years). In addition, the prevalence of hypertension was significantly (*p* = 0.002) higher in patients who died (78.9%) compared to the survivors (65.5%). Similar results were noted for coronary heart disease (36.6% vs. 24.1%, *p* = 0.002), myocardial infarction (17.6% vs. 10.7%, *p* = 0.024), heart failure (26.8% vs. 8.8%, *p* < 0.001), peripheral artery disease (26.8% vs. 10.9%, *p* < 0.001), chronic kidney disease (36.6% vs 17.9%, *p* < 0.001), and cancer (19.7% vs. 10.3%, *p* = 0.002).

### 3.2. Biomarker of COVID-19 Mortality

**Figure 1:**
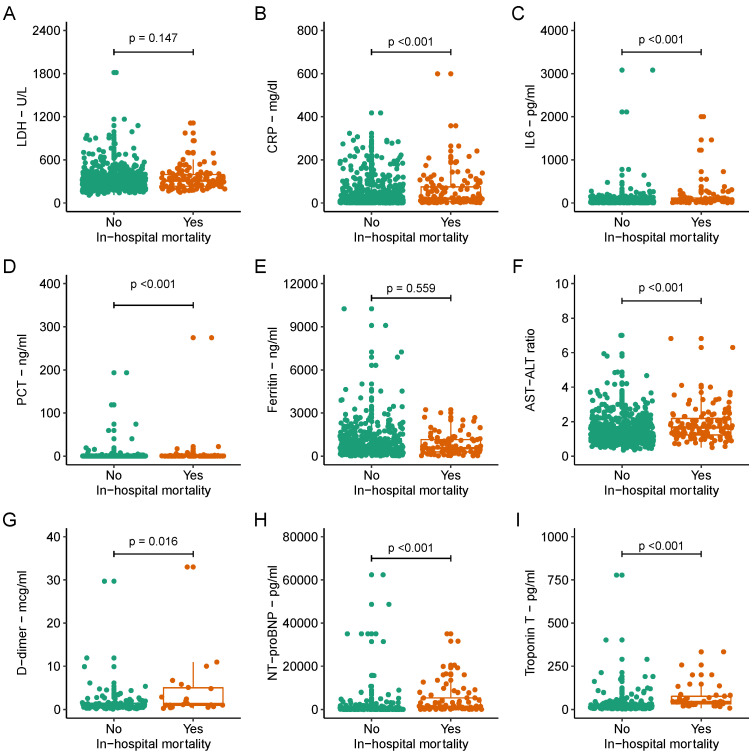
Distribution of biomarkers by in-hospital mortality. Each subplot (**A**–**I**) compares the distribution of biomarker between patients who died in the hospital and those who survived. Y-axis represents the range of values for each biomarker and X-axis represents patients who died versus those who were alive. LDH: lactate dehydrogenase; CRP: C-reactive protein; IL6: interleukin 6; PCT: procalcitonin; AST: aspartate aminotransferase; ALT: alanine aminotransferase; NT-proBNP: N-terminal pro-brain natriuretic peptide. *p* = Wilcoxon rank-sum test *p*-value.

The median [IQR] values of biomarkers and their comparison by in-hospital mortality status are shown in Table 1 and Figure 1. For inflammatory markers, the median value of LDH was 288.0 U/L [160.0], CRP was 12.1 mg/dL [43.4], IL-6 was 41.8 pg/mL [56.8], PCT was 0.1 ng/mL [0.1], and ferritin was 568.0 ng/mL [938.0]. The median AST–ALT ratio was 1.3 [0.8], and coagulation marker D-dimer was 1.0 mcg/mL [1.0]. For cardiac markers, the median level of NT-proBNP was 418.5 pg/mL [1464.0], and Troponin T was 20.0 [31.0] pg/mL.

Comparison of biomarkers with in-hospital mortality shows that the median levels of CRP (20.4 [66.9] vs. 10.7 [34.2], *p* < 0.001), IL-6 (67.7 [80.7] vs. 38.5 [49.3], *p* < 0.001), and PCT (0.2 [0.4] vs. 0.1 [0.1], *p* < 0.001) were significantly higher in patients who died in the hospital compared to those who survived. However, the median levels of LDH (311.5 [165.0] vs. 281.0 [159.0], *p* = 0.147) and ferritin (562.0 [864.0] vs. 570.0 [944.0], *p* = 0.559) did not differ significantly between non-survivors and survivors. For hepatic markers, the median levels of AST (42.0 [34.5] vs. 36.0 [28.0], *p* = 0.027), ALT (27.0 [20.0] vs. 29.0 [27.0], *p* = 0.037), and AST–ALT ratio (1.7 [1.0] vs. 1.3 [0.7], *p* < 0.001) were significantly higher in non-survivors versus survivors. The median level of coagulation marker D-dimer (1.3 [4.1] vs. 0.9 [1.0], *p* < 0.001) was significantly higher in people who died compared to those who survived. Similar results were observed for the cardiac markers NT-proBNP (1333.5 [5003.5] vs. 297.0 [730.0], *p* < 0.001) and Troponin T (43.0 [44.0] vs. 16.0 [22.0], *p* < 0.001) (Figure 1).

### 3.3. Correlation between Biomarkers

**Figure 2:**
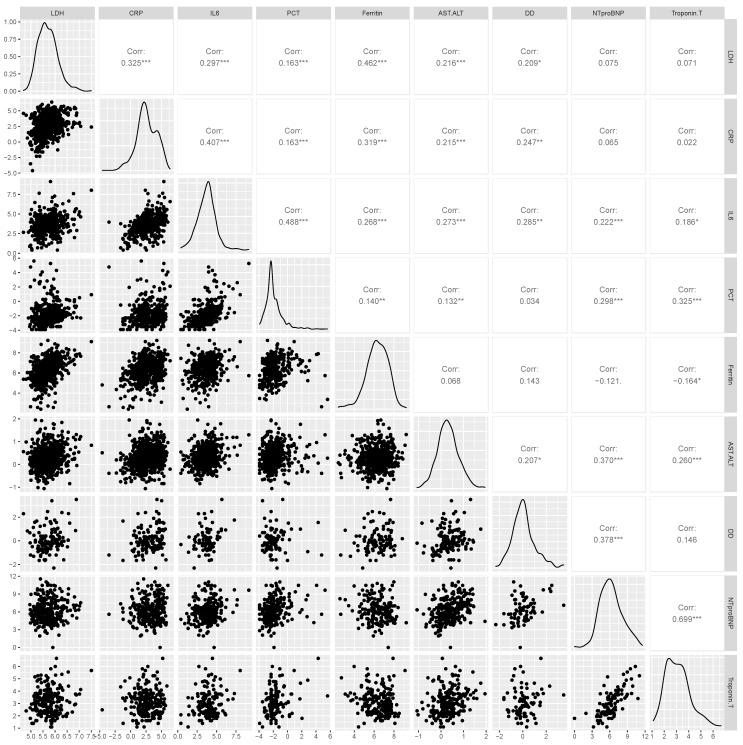
Pearson correlation plots of log-transformed biomarkers. X and Y labels correspond to log-transformed values of biomarkers. Corr: corresponds to Pearson correlation coefficient. LDH: lactate dehydrogenase; CRP: C-reactive protein; IL6: interleukin 6; PCT: procalcitonin; AST: aspartate aminotransferase; ALT: alanine aminotransferase; NT-proBNP: N-terminal pro-brain natriuretic peptide. * The correlation is significant at < 0.05. ** The correlation is significant at < 0.01. *** The correlation is significant at < 0.001.

Figure 2 shows the Pearson correlation analyses between biomarkers. IL-6 had a moderate positive correlation with all other biomarkers. NT-proBNP had a strong positive correlation with Troponin T (r = 0.70, *p* < 0.001), a weak positive correlation with IL-6 (r = 0.22, *p* = < 0.001), and a moderate positive correlation with PCT (r = 0.30, *p* < 0.001), AST–ALT ratio (r = 0.37, *p* < 0.001), and D-dimer (r = 0.38, *p* < 0.001). Ferritin was moderately positively correlated with LDH (r = 0.46, *p* < 0.001) and CRP (r = 0.32, *p* < 0.001). There was a moderate positive correlation between PCT, NT-proBNP, Troponin T, and IL6. LDH was moderately positively correlated with every biomarker except NT-proBNP and Troponin T. The AST–ALT ratio was moderately positively correlated with other biomarkers, except ferritin and PCT. D-Dimer was moderately positively correlated with all biomarkers except PCT and ferritin.

### 3.4. Association of Biomarkers with In-Hospital Mortality

In simple logistic regression analysis, CRP, IL-6, PCT, D-dimer, AST–ALT ratio, NT-proBNP, and Troponin T were significantly associated with in-hospital mortality (Table 2). In multiple logistic regression analysis, which was adjusted for age, sex, and type of diabetes mellitus, CRP, IL-6, PCT, AST–ALT ratio, NT-proBNP, and Troponin T remained significantly associated with in-hospital mortality. While LDH became a significant predictor of mortality in the multiple logistic regression analysis, D-dimer did not retain significance in the multiple logistic regression analysis. Ferritin was not significantly associated with in-hospital mortality in either simple or multiple logistic regression analysis.

### 3.5. Predictive Performance of Biomarkers

**Figure 3:**
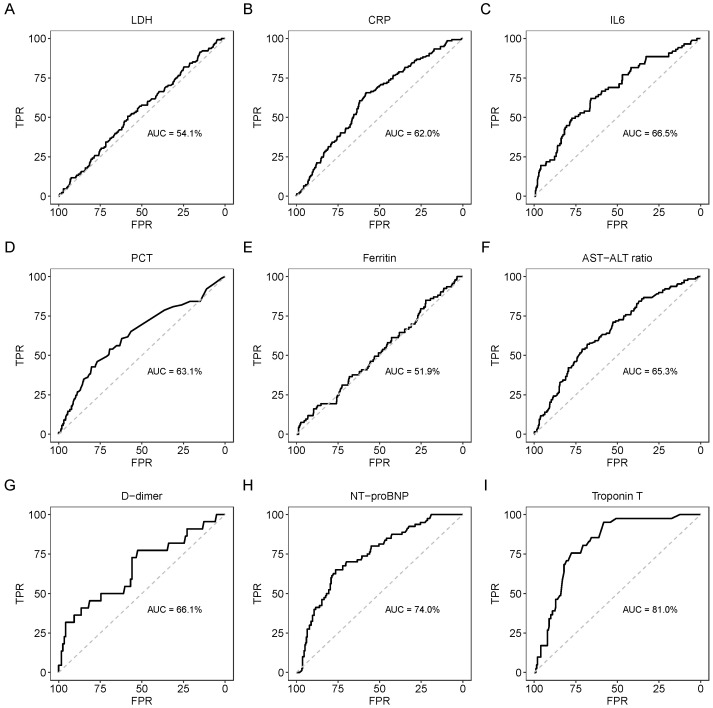
ROC curve plots of biomarkers with in-hospital mortality. In each subplot (**A**–**I**), grey diagonal line shows no predictive value and black line shows the actual predictive curve for each biomarker. Y axis represents TPR while X-axis represents FPR. AUC: area under the curve; TPR: true positive rate; FPR: false positive rate; LDH: lactate dehydrogenase; CRP: C-reactive protein; IL6: interleukin 6; PCT: procalcitonin; AST: aspartate aminotransferase; ALT: alanine aminotransferase; NT-proBNP: N-terminal pro-brain natriuretic peptide.

NT-proBNP showed good discrimination with an AUC of 0.74, and Troponin T showed excellent discrimination with an AUC of 0.81 (Figure 3), while all other investigated biomarkers did not show good discrimination. NT-proBNP showed good calibration too (H-L statistics = 9.50, *p* = 0.302), whereas Troponin T showed poor calibration (H-L statistics = 20.03, *p* = 0.010) (Table 3).

## 4. Discussion

In this multicenter retrospective cohort analysis of people with prediabetes and diabetes mellitus hospitalized with COVID-19, we assessed the performance of various inflammation, hepatic, coagulation, and cardiac biomarkers for predicting in-hospital mortality. Of the total number of hospitalized individuals, 70.8% had type 2 diabetes mellitus, 5.8% had type 1 diabetes mellitus, 14.9% had prediabetes, and 8.6% had other types of diabetes mellitus, and a total of 19.0% of patients died in the hospital. Of the studied biomarkers, the cardiac biomarkers NT-proBNP (74.0%) and Troponin T (81.0%) showed satisfactory predictive performance for in-hospital mortality, while none of the inflammatory, hepatic, or coagulation markers demonstrated a satisfactory predictive performance in our cohort.

In this study, the in-hospital mortality was 19.0% in patients with prediabetes and diabetes mellitus. Compared to our findings, Hammad et al. reported a higher in-hospital mortality rate of 38.2% in patients with diabetes mellitus, while Acharya et al. reported a similar mortality rate of 20.0% in patients with diabetes mellitus compared to 7.4% in the whole population [16,17]. In contrast with other studies, the in-hospital mortality was not significantly different between males and females in our analysis; however, the populations being hospitalized are not directly comparable between countries with different health care systems [17,18].

Our analysis showed significantly higher NT-proBNP and Troponin T levels in patients who died in the hospital compared to those who survived. In addition, the elevated levels of these cardiac biomarkers were significantly associated with increased in-hospital mortality. These associations remained significant even after excluding patients with a history of heart failure and adjusting for other comorbidities, which further confirms the independent association of cardiac biomarkers with mortality in our cohort. Only a few other studies have investigated the role of biomarkers with adverse outcomes in patients with diabetes mellitus following a COVID-19 infection. Reviews by Ceriello et al., Lippi et al., Bavishi et al., and Lavie et al. highlighted the importance of measuring NT-proBNP and Troponin to aid in the diagnosis and prognostication of COVID-19 in patients with and without diabetes mellitus because of the high risk of cardiac injury in this population [10,11,19,20]. A recent meta-analysis also showed increased levels of Troponin in people with severe COVID-19 infection [21]. Another study from China comprising 28 patients with diabetes mellitus and COVID-19 also reported that NT-proBNP was significantly increased in ICU patients compared to non-ICU patients [22]. Studies assessing the predictive performance of cardiac biomarkers in patients with diabetes mellitus are scarce; however, Cunningham et al. showed the good predictive performance of NT-proBNP (area under the ROC curve: 0.75) in patients with COVID-19 [23]. Various pathophysiological mechanisms have been identified to alter cardiac markers in COVID-19. It is postulated that viral illnesses such as COVID-19 can damage myocardial cells, especially in patients with pre-existing cardiovascular diseases, through mechanisms such as systemic inflammatory responses, direct damage by the virus, and hypoxia [24]. Hypoxia induces pulmonary hypertension, which subsequently increases ventricular wall stress and thus releases NT-proBNP [25].

Although most inflammatory biomarkers (CRP, LDH, IL6, PCT, and ferritin) were significantly associated with in-hospital mortality, none of these biomarkers demonstrated satisfactory predictive performance in our study. Jayanthy and colleagues evaluated the predictive performance of CRP in patients with COVID-19 and a subgroup of diabetes mellitus patients and demonstrated a discrimination of 0.72 in this subgroup [26]. In another study conducted by Wang and colleagues comprising patients with diabetes mellitus, LDH, PCT, CRP, ferritin, and IL-6 were significantly increased in ICU patients compared to non-ICU patients [22]. However, they did not evaluate the predictive performance of these biomarkers. Hammad and colleagues showed abnormal albeit insignificant LDH and CRP levels in their patients (*n* = 118) with diabetes mellitus [17], while Bhatti and colleagues, in their analysis of 103 patients with diabetes mellitus and prediabetes with COVID-19, showed significantly higher levels of ferritin and CRP in patients who needed ICU care [18]. The discrepancies in the findings regarding the predictive role of inflammatory markers in patients with diabetes warrant further studies in larger patient cohorts.

The coagulation biomarker d-dimer was significantly associated with in-hospital mortality; however, its discrimination was unsatisfactory (0.66) in our cohort. In comparison, Miri and colleagues reported an AUC of 0.75 for d-dimer in COVID-19 patients with diabetes [10]. Furthermore, the study by Wang and colleagues showed D-dimer to be significantly increased in ICU patients compared to non-ICU patients with diabetes mellitus. Likewise, Bhatti and colleagues showed significantly higher levels of d-dimer in patients who needed ICU care. However, these studies did not explore the predictive role of d-dimer [18,22]. The association between elevated d-dimer and in-hospital mortality in patients with diabetes mellitus could be explained by hyperglycemia-related alterations in the thrombosis cascade [27].

For hepatic biomarkers, we analyzed only the AST–ALT ratio and found it to be significantly associated with in-hospital mortality, albeit with inadequate predictive performance. Hammad and colleagues also showed increased ALT to be a significant positive predictor of mortality in patients with diabetes mellitus and COVID-19 [17]. Elevated liver enzymes indicate liver damage, which could result from immune-related injury or direct liver cytotoxicity. However, liver-damaging medications, such as some antivirals and antibiotics, which are used in the treatment of COVID-19, could also elevate liver enzymes [28].

Our study is not without limitations. One limitation is the unavailability of comparison data for patients without diabetes mellitus who were hospitalized with COVID-19. Another limitation is the limited number of people with prediabetes in our study. For this reason, we could not perform a comparative analysis between patients with prediabetes and diabetes mellitus. As often encountered in routinely collected health data, the classification of diabetes mellitus into subtypes may not be accurate. In addition, due to the pragmatic design of this study, laboratory data were not available for all patients. Furthermore, biomarkers were measured only at the time of admission, which may not fully reflect the clinical course and prognosis of COVID-19.

The major strengths of the study are its adequate sample size, the longitudinal study design, and the inclusion of multiple hospitals across Austria. The adjustment for known confounding factors to determine the independent association of biomarkers with in-hospital mortality is another strength of our study.

## 5. Conclusions

In conclusion, the cardiac biomarkers Troponin T and NT-proBNP demonstrated satisfactory predictive performance in people with prediabetes and diabetes in this study. As these biomarkers can be routinely measured in the clinical setting, they could therefore be used as prognostic biomarkers for COVID-19 patients. However, we recommend more studies investigating the prognostic role of these biomarkers in larger diabetes cohorts.

## Figures and Tables

**Table 1:** Comparison of characteristics, comorbidities, and biomarkers with in-hospital mortality in patients with prediabetes and diabetes mellitus hospitalized with COVID-19.

Variables	*n*	All	In-Hospital Mortality		*p*-Value
			Yes	No	
All, *n* (%)	747	--	142 (19.0)	605 (81.0)	--
Characteristics					
Age—years, mean ± SD	717	70.3 ±13.3	78.63 ±10.0	68.3 ±13.2	<0.001
Sex, *n* (%)	747				
Male		518 (69.3)	95 (66.9)	423 (69.9)	0.483
Female		229 (30.7)	47 (33.1)	182 (30.1)	
Smoking status, *n* (%)	747				
Non-smoker		372 (49.8)	70 (49.3)	302 (49.9)	0.399
Former smoker		97 (13.0)	24 (16.9)	73 (12.1)	
Current smoker		23 (3.1)	5 (3.5)	18 (3.0)	
Unknown		255 (34.1)	43 (30.3)	212 (35.0)	
Body mass index—kg/m^2^, mean ± SD	390	29.0 ±5.9	29.52 ±6.7	28.9 ±5.7	0.439
Type of diabetes mellitus, *n* (%)	747				
Prediabetes		111 (14.9)	12 (8.5)	99 (16.4)	0.010
Type 1 diabetes mellitus		43 (5.8)	5 (3.5)	38 (6.3)	
Type 2 diabetes mellitus		529 (70.8)	117 (82.4)	412 (68.1)	
Other diabetes mellitus		64 (8.6)	8 (5.6)	56 (9.3)	
Comorbidities					
Hypertension, *n* (%)	747	508 (68.0)	112 (78.9)	396 (65.5)	0.002
Coronary heart disease, *n* (%)	747	198 (26.5)	52 (36.6)	146 (24.1)	0.002
Myocardial infarction, *n* (%)	747	90 (12.1)	25 (17.6)	65 (10.7)	0.024
Heart failure, *n* (%)	747	91 (12.2)	38 (26.8)	53 (8.8)	<0.001
Peripheral artery disease, *n* (%)	747	104 (13.9)	38 (26.8)	66 (10.9)	<0.001
Stroke, *n* (%)	747	57 (7.6)	16 (11.3)	41 (6.8)	0.070
Chronic kidney disease, *n* (%)	747	160 (21.4)	52 (36.6)	108 (17.9)	<0.001
Cancer, *n* (%)	747	90 (12.1)	28 (19.7)	62 (10.3)	0.002
Respiratory disease, *n* (%)	747	147 (19.7)	35 (24.7)	112 (18.5)	0.098
Liver disease, *n* (%)	747	57 (7.6)	14 (9.9)	43 (7.1)	0.266
Inflammatory biomarkers					
LDH—U/L, median [IQR]	681	288.0 [160.0]	311.5 [165.0]	281.0 [159.0]	0.147
CRP—mg/dL, median [IQR]	711	12.1 [43.5]	20.4 [66.9]	10.7 [34.2]	<0.001
IL6—pg/mL, median [IQR]	489	41.8 [56.8]	67.7 [80.7]	38.5 [49.3]	<0.001
PCT—ng/mL, median [IQR]	503	0.1 [0.1]	0.2 [0.4]	0.1 [0.1]	<0.001
Ferritin—ng/mL, median [IQR]	555	568.0 [938.0]	562.0 [864.0]	570.0 [944.0]	0.559
Hepatic biomarkers					
AST—U/L, median [IQR]	565	38.0 [29.0]	42.0 [34.5]	36.0 [28.0]	0.027
ALT—U/L, median [IQR]	578	29.0 [25.0]	27.0 [20.0]	29.0 [27.0]	0.037
AST–ALT ratio	564	1.33 [0.8]	1.67 [1.0]	1.28 [0.7]	<0.001
Coagulation biomarkers					
D-dimer—mcg/mL, median [IQR]	140	0.99 [0.97]	1.28 [4.08]	0.90 [0.98]	0.016
Cardiac biomarkers					
NT-proBNP—pg/mL, median [IQR]	296	418.5 [1464.0]	1333.5 [5003.5]	297.0 [730.0]	<0.001
Troponin T—pg/mL, median [IQR]	242	20.0 [31.0]	43.0 [44.0]	16.0 [22.0]	<0.001

LDH: lactate dehydrogenase; CRP: C-reactive protein; IL6: interleukin 6; PCT: procalcitonin; AST: aspartate aminotransferase; ALT: alanine transaminase; NT-proBNP: N-terminal pro-B-type natriuretic peptide.

**Table 2:** Logistic regression analysis of biomarkers with in-hospital mortality.

Biomarkers	Simple Logistic Regression			Multiple Logistic Regression		
	OR	95%CI	*p*-Value	AOR	95%CI	*p*-Value
Inflammatory biomarkers						
LDH—U/L	1.4	0.88–2.25	0.158	2.03	1.21–3.42	0.008
CRP—mg/dL	1.3	1.15–1.47	<0.001	1.33	1.16–1.52	<0.001
IL6—pg/mL	1.66	1.34–2.06	<0.001	1.6	1.27–2.01	<0.001
PCT—ng/mL	1.31	1.13–1.51	<0.001	1.25	1.06–1.48	0.007
Ferritin—ng/mL	0.9	0.74–1.10	0.3	1.07	0.86–1.35	0.541
Coagulation biomarkers						
D-dimer—mcg/mL	1.93	1.22–3.03	0.005	1.66	0.97–2.82	0.063
Hepatic biomarkers						
AST–ALT ratio	3	1.97–4.56	<0.001	1.89	1.19–3.01	0.007
Cardiac biomarkers						
NT-proBNP—pg/mL	1.59	1.35–1.86	<0.001	1.5	1.24–1.80	<0.001
Troponin T—pg/mL	2.78	1.90–4.07	<0.001	2.2	1.44–3.35	<0.001

LDH: lactate dehydrogenase; CRP: C-reactive protein; IL6: interleukin 6; PCT: procalcitonin; AST: aspartate aminotransferase; ALT: alanine aminotransferase; NT-proBNP: N-terminal pro-brain natriuretic peptide. Multiple logistic regression model was adjusted for age, sex, and type of diabetes mellitus.

**Table 3:** Hosmer–Lemeshow goodness-of-fit test of biomarkers.

Biomarkers	Hosmer–Lemeshow Test	
	Statistics	*p*-Value
Inflammatory biomarkers		
LDH—U/L	3.22	0.920
CRP—mg/dL	5.82	0.667
IL6—pg/mL	3.81	0.874
PCT—ng/mL	6.63	0.577
Ferritin—ng/mL	6.42	0.600
Coagulation biomarkers		
D-dimer—mcg/mL	6.19	0.626
*Hepatic biomarkers*		
AST–ALT ratio	7.96	0.437
Cardiac biomarkers		
NT-proBNP—pg/mL	9.50	0.302
Troponin T—pg/mL	20.03	0.010

LDH: lactate dehydrogenase; CRP: C-reactive protein; IL6: interleukin 6; PCT: procalcitonin; AST: aspartate aminotransferase; ALT: alanine aminotransferase; NT-proBNP: N-terminal pro-brain natriuretic peptide.

## Data Availability

The data used in this study are owned by the Austrian Diabetes Association and can be provided upon request.

## Supplementary Materials

The following supporting information can be downloaded at: https://www.mdpi.com/article/10.3390/v14061285/s1.
