# Supplementary material for: Biomarkers Predictive for In-Hospital Mortality in Patients with Diabetes Mellitus and Prediabetes Hospitalized for COVID-19 in Austria: An Analysis of COVID-19 in Diabetes Registry
Source: Viruses. 2022 Jun 13;14(6):1285. doi: 10.3390/v14061285 (PMC9229324; doi:10.3390/v14061285)
Supplement: Supplementary file 1 [file viruses-14-01285-s001.zip › viruses-1765438-supplementary/COVID-19 in Austria Study Group.pdf]

Members of the “COVID-19 in Diabetes in Austria Study Group

Harald Sourij, Norbert J. Tripolt, Caren Sourij, Farah Abbas, Oliver Malle, Julia Mader
Medical University of Graz, Austria

Peter Fasching, Gersina Rega-Kaun, Kadriye Aydinkov-Tuzcu, Alexander Bräuer, Brigitte Bernhardt
Medical Division for Endocrinology, Rheumatology, and Acute Geriatrics, Wilhelminen Hospital Vienna, Austria

Christian Ciardi, Marc Schaber, Anna Schapfl, David Fiegl
Clinical Division for Internal Medicine, Endocrinology, Diabetology, and Metabolic Diseases St. Vinzenz Hospital Zams, Austria

Martin Clodi, Carmen Klammer, Michael Resl, Matthias Heinzl, Roland Feldbauer, Johannes Pohlhammer
Clinical Division for Internal Medicine, Konventhospital Barmherzige Brüder Linz, Austria

Mario Karolyi, Erich Pawelka
4th Medical Division with Infectiology, SMZ Süd-KFJ-Hospital, Vienna, Austria

Alexandra Kautzky-Willer, Peter Wolf
Clinical Division for Endocrinology and Diabetology and Metabolic Diseases, AKH Vienna, Austria

Lars Stechemesser, Michael Schranz
Department for Internal Medicine I, Paracelsus Medical University, Salzburg, Austria

Harald Stingl, Michael Wagner, Reinhard Würfel
Clinical Division for Internal Medicine, Hospital Melk, Austria

Thomas M. Stulnig, Slobodan Peric, Andreas Zitterl
3rd Medical Department and Karl Landsteiner Institute for Metabolic Diseases and Nephrology, Clinic Hietzing, Vienna Health Care Group, Austria

Susanne Kaser, Claudia Ress
Department for Internal Medicine I, Medical University Innsbruck, Austria
